# Depressive symptoms among patients with cancer receiving palliative care: a cross-sectional study

**DOI:** 10.3389/fpsyg.2026.1959368

**Published:** 2026-10-28

**Authors:** Belal Sharaf, Hanan Jawa, Shady Kamel

**Affiliations:** 1 Palliative Care Department, King Faisal Specialist Hospital and Research Centre, Jeddah, Saudi Arabia; 2 Research Center, King Faisal Specialist Hospital and Research Centre, Jeddah, Saudi Arabia

**Keywords:** cancer, depressive symptoms, palliative care, PHQ-9, psychological screening, Saudi Arabia

## Abstract

**Background:**

Depressive symptoms are clinically important in palliative oncology, but interpretation is complicated by overlap between cancer-related somatic symptoms and depression-screening items. Saudi studies report psychological distress in advanced cancer, but PHQ-9 severity and symptom patterns in palliative oncology remain less characterized.

**Objective:**

To characterize the frequency and severity of depressive symptoms using the Arabic PHQ-9, describe individual symptom patterns including thoughts of death or self-harm, and explore selected demographic and clinical characteristics associated with a PHQ-9 score ≥10.

**Methods:**

This single-center cross-sectional study recruited adults with cancer receiving palliative care over 12 months. Data were collected through interviews and medical-record review. PHQ-9 severity categories and screening thresholds were summarized descriptively. Exploratory comparisons of PHQ-9 < 10 versus ≥10 used Fisher’s exact tests, unadjusted odds ratios (ORs) with 95% confidence intervals (CIs), and the Mann–Whitney *U* test for age. Individual cancer types and individual comorbidities were descriptive only.

**Results:**

Of 150 patients approached, 90 consented, were assessed as eligible and participated (60.0%); eligibility was unknown for the 60 who declined. PHQ-9 scores were none/minimal in 43 (47.8%), mild in 22 (24.4%), moderate in 6 (6.7%), moderately severe in 9 (10.0%) and severe in 10 (11.1%). Scores ≥5, ≥10 and ≥15 occurred in 47 (52.2%; 95% CI 42.0–62.2%), 25 (27.8%; 19.6–37.8%) and 19 (21.1%; 14.0–30.6%), respectively. All 13 participants endorsing thoughts of death or self-harm (14.4%; 8.6–23.2%) received same-day psychiatric assessment. Exploratory associations were imprecise, with all categorical OR confidence intervals including 1.

**Conclusion:**

Approximately half of participants reported at least mild depressive symptoms, while more than one-quarter met the conventional moderate-or-greater PHQ-9 screening threshold. Item-level assessment identified clinically important symptom burden, including thoughts of death or self-harm. These findings support careful early recognition and individualized assessment rather than diagnostic interpretation of screening scores.

## Introduction

Depressive symptoms are common among patients with advanced cancer and may compound psychological distress, functional impairment, and difficulty coping with serious illness. Estimates vary substantially according to disease stage, care setting, assessment instrument, cutoff point, and whether a diagnostic interview is used. These distinctions are particularly important in palliative care, where symptom burden is high and the goals of assessment include recognition of potentially treatable psychological distress while minimizing additional burden on patients (Ikhile et al., 2024; Kulpa et al., 2023; Gontijo Garcia et al., 2023).

The Patient Health Questionnaire-9 (PHQ-9) is a validated screening instrument that grades depressive symptom severity but does not independently establish a depressive disorder. Scores of 5–9 indicate mild symptoms, whereas a score of 10 or greater is commonly used as a threshold for moderate-or-greater symptoms warranting further clinical assessment (Grapp et al., 2019; Kroenke et al., 2001). Screening estimates therefore should not be interpreted as the prevalence of clinically diagnosed depression.

Assessment is particularly challenging in advanced cancer because several PHQ-9 items overlap with manifestations of cancer and its treatment. Fatigue, sleep disturbance, appetite change, impaired concentration, and psychomotor change may arise from depression, cancer-related symptom burden, treatment toxicity, or combinations of these factors (Grapp et al., 2019; Brown and Kroenke, 2009). Item-level interpretation and clinical context are therefore important alongside the total score.

Saudi evidence is growing but remains heterogeneous. Recent studies in advanced or palliative cancer populations have used instruments such as the Hospital Anxiety and Depression Scale (HADS), while a recent study in older Saudi adults receiving palliative oncology care used the PHQ-9 in conjunction with patient activation and quality-of-life measures (Alotaibi and Alsuhail, 2025; Bakhsh et al., 2024; Ibrahim and Zaghamir, 2026). Differences in population, instrument, threshold, and clinical context limit direct comparison. Accordingly, the contribution of the present study is not geographic primacy, but detailed characterization of the full PHQ-9 severity spectrum and individual symptom pattern within a Saudi tertiary palliative oncology service, including separate reporting of thoughts of death or self-harm and a linked clinical safety pathway.

The primary objective was to characterize the frequency and severity of depressive symptoms using the previously validated Arabic PHQ-9 among patients with cancer receiving palliative care who were not under active psychiatric treatment. Secondary objectives were to describe item-level symptom patterns, including PHQ-9 item 9, and to explore selected demographic and clinical characteristics associated with a PHQ-9 score ≥10. This case-finding approach was intended to support earlier recognition and targeted clinical assessment of psychological distress rather than to diagnose depressive disorder or demonstrate that screening prevents depression.

## Subjects and methods

### Study design and setting

A cross-sectional descriptive study was conducted among adult patients with cancer receiving care through the palliative care service at King Faisal Specialist Hospital & Research Centre, Jeddah, Saudi Arabia, from 31 May 2025 to 31 May 2026. The study is reported in accordance with the Strengthening the Reporting of Observational Studies in Epidemiology (STROBE) statement.

### Participants and recruitment

Participants were recruited from the outpatient palliative clinic and home healthcare service. The sampling frame comprised all 150 patients receiving palliative care during the study period, all of whom were approached. Recruitment setting was not retained as a participant-level analytic variable; therefore, setting-specific counts and comparisons could not be reconstructed.

### Eligibility criteria

Eligible participants were adults with a confirmed cancer diagnosis who could understand the study information, communicate a voluntary decision and provide their own questionnaire responses. Patients with severe cognitive impairment or receiving active psychiatric treatment were excluded. Medical records were reviewed for cognitive impairment and clinical history, and the recruiting clinician assessed the ability to communicate and provide consent. The investigator could clarify questionnaire instructions or wording without selecting or suggesting responses.

The exclusion of patients receiving active psychiatric treatment reflected the case-finding purpose of identifying potentially unrecognized symptoms among patients not already in an active psychiatric pathway. Because treatment may modify current symptom scores, these patients were excluded from this specific screening cohort.

### Sample size and participant flow

No formal prospective sample-size calculation was performed. The sample was determined by the fixed one-year recruitment period and participation. Of 150 patients approached, 60 declined before formal eligibility assessment; their eligibility status was unknown. Reported reasons were lack of time, feeling well, perceiving no need to participate, or no reason provided. Counts by reason and clinical characteristics of non-participants were unavailable. Ninety patients consented and were assessed for eligibility. All met the eligibility criteria, none were excluded, and all 90 were included in the analysis (60.0% of patients approached; Figure 1).

**Figure 1:**
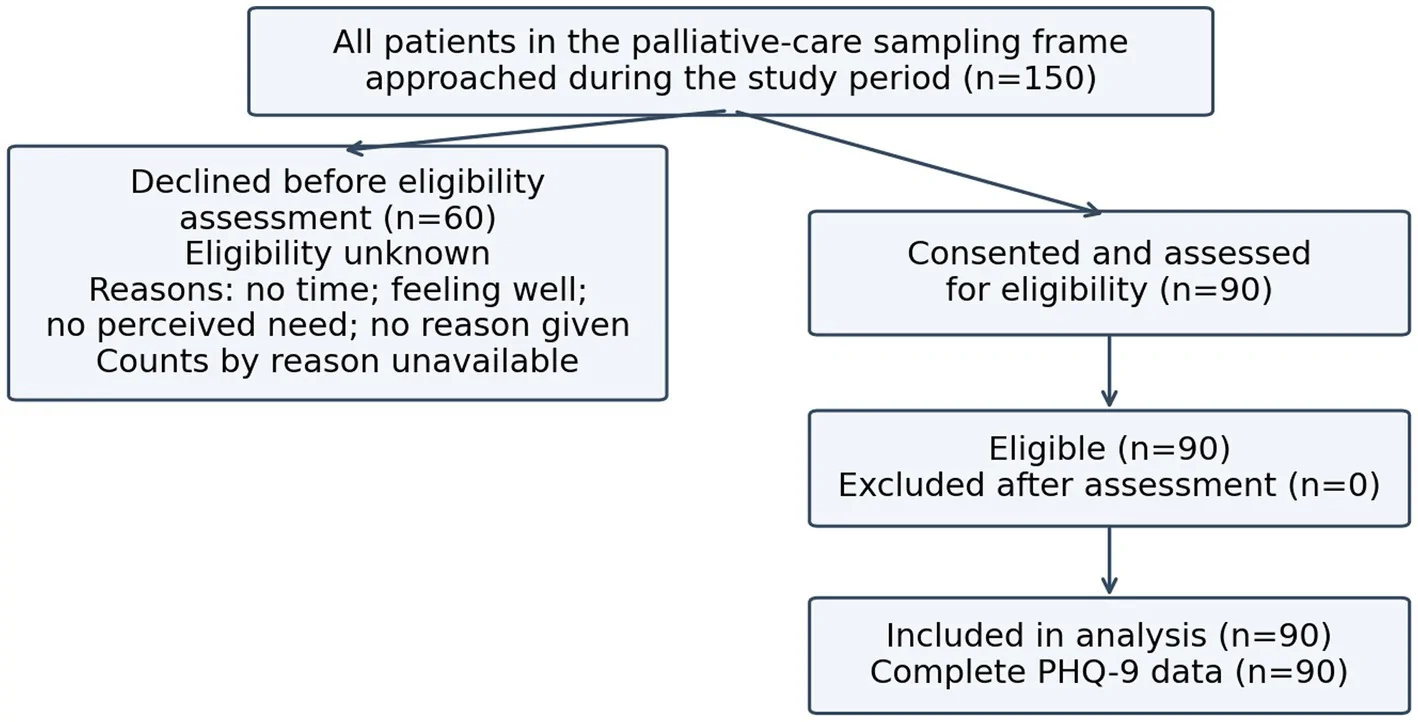
Participant flow through recruitment and analysis.

### PHQ-9 and safety procedure

The Arabic-language PHQ-9 was administered through participant interviews and assessed symptoms over the preceding 2 weeks. Each item was scored from 0 to 3, giving a total score of 0–27. Scores were categorized as none/minimal (0–4), mild (5–9), moderate (10–14), moderately severe (15–19), or severe (20–27). Scores ≥5, ≥10 and ≥15 were reported separately to describe at least mild, moderate-or-greater and moderately severe-or-greater depressive symptoms, respectively. These cumulative thresholds overlap and represent screening categories rather than clinical diagnoses (Grapp et al., 2019; Kroenke et al., 2001; AlHadi et al., 2017).

All nine PHQ-9 items were complete for all 90 participants, and their sum matched the stored total score for every participant. Item 9 was described as thoughts of death or self-harm because endorsement does not itself establish suicidal intent or diagnosis of suicidality. Any endorsement (score ≥1) prompted immediate referral to the Emergency Department urgent clinic for same-day psychiatric assessment. Clinical assessment distinguished passive wishes for death from active suicidal thoughts and explored intent, plans, access to means, previous attempts, contributing physical and psychological symptoms, and decision-making capacity. Management addressed identified contributors to distress and was individualized to clinical condition and goals of care. Patients without immediate safety concerns received psychological, psychiatric and palliative support as indicated. Active intent or inability to maintain safety required immediate protective measures and continuous supervision, with escalation to an appropriate supervised care setting when safety could not be maintained in the current setting. Assessment findings and management were documented in the medical record and verified by the clinical teams. Figure 2 summarizes this local clinical pathway. Clinical follow-up continued as indicated by the treating teams; however, subsequent clinical outcomes were not systematically collected for this study.

**Figure 2:**
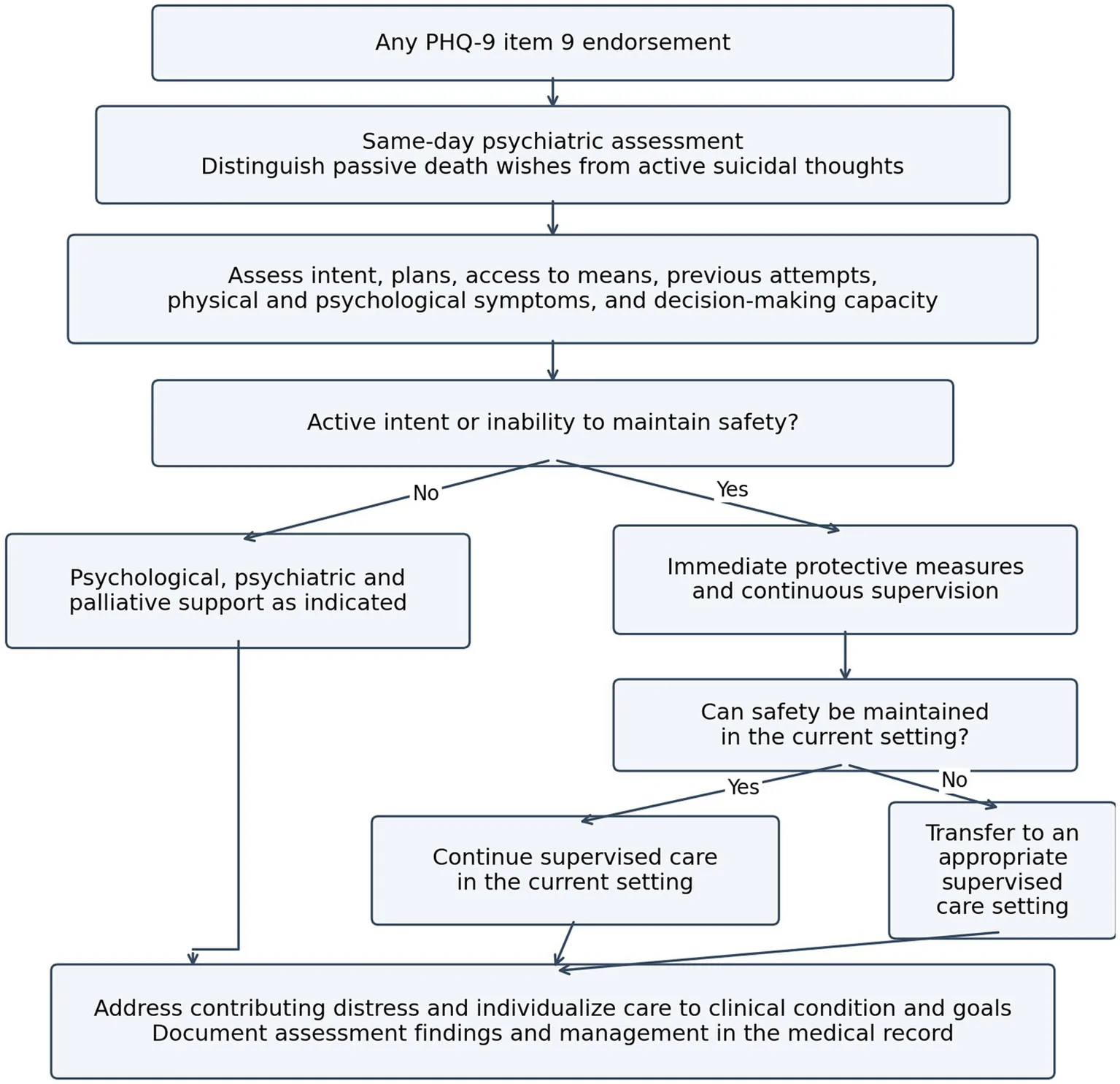
Clinical response pathway following PHQ-9 item 9 endorsement. The pathway includes contingency actions for immediate safety concerns. No immediate suicide or self-harm risk was identified among the 13 assessed participants. Clinical teams verified assessment findings and management using psychiatric records.

### Consent and capacity

The Institutional Review Board waived written consent and formally approved verbal consent. Before enrollment, the recruiting clinician confirmed that the patient was conscious, understood the study information, could communicate a voluntary decision, and could complete the questionnaire. The principal investigator documented verbal consent in a signed consent log.

### Data sources and variable definitions

Data were obtained through participant interviews and medical-record review. Smoking, education, marital status, employment and PHQ-9 responses were collected directly from participants. Medical records were reviewed to obtain or verify age, sex, cancer diagnosis, metastatic status, do-not-resuscitate status, comorbidities, family psychiatric history, treatment and cognitive impairment before the final dataset was assembled. Psychiatric assessment completion, findings and management following item 9 endorsement were verified by the clinical teams using psychiatric records. Demographic and clinical information reflected the patient’s status at the time of the study interview. Previous cancer surgery and family psychiatric history were recorded as historical variables, while PHQ-9 responses covered the preceding 2 weeks.

Age was recorded in years and sex as female or male. Marital status was analyzed as currently married versus not married. Employment indicated currently holding a job versus not holding a job, irrespective of ability to work; retirement, unemployment and illness-related inability to work were not distinguished. Smoking included regular or occasional smoking; non-smoking was defined as no smoking during the preceding 6 months. Education represented the highest completed level: low (no formal education or below preparatory level), middle (preparatory), high (high school), or university or above.

Cancer diagnosis was grouped as colon, breast, lung, hematological or other cancer. Disease extent was recorded as local or metastatic; metastatic disease meant spread to distant organs or distant lymph nodes. DNR indicated a documented do-not-resuscitate decision at assessment. Chemotherapy, radiation therapy, hormonal therapy and immunotherapy were recorded as having been received at any time up to the study interview, including previous or ongoing treatment. Surgery indicated any previous cancer-related surgery. Treatment categories were not mutually exclusive. Hypertension, diabetes, cardiac disease, chronic obstructive pulmonary disease and asthma were recorded as documented comorbidities rather than newly diagnosed using study-specific tests. Specific cardiac diagnoses were not distinguished. Any comorbidity indicated at least one of these five conditions. Family psychiatric history indicated a recorded family history of psychiatric illness; the affected relative and disorder type were not specified. Individual cancer types, educational categories and individual comorbidities were summarized descriptively only.

### Statistical analysis

Statistical analyses were performed using IBM SPSS Statistics version 31.0 (IBM Corp., Armonk, NY, USA), with independent verification of the revised analyses. Categorical variables are presented as frequencies and percentages. Age and total PHQ-9 score are summarized using the median and interquartile range (IQR). Wilson 95% confidence intervals are reported for PHQ-9 thresholds ≥5, ≥10, and ≥15 and for item 9 endorsement. The five PHQ-9 severity categories were retained for descriptive presentation. For exploratory analysis, participants were grouped as PHQ-9 < 10 (*n* = 65) versus ≥10 (*n* = 25). Binary categorical variables were compared using two-sided Fisher’s exact tests, with unadjusted ORs and 95% CIs. Age was compared using the Mann–Whitney *U* test. Individual cancer types and individual comorbidities were not tested inferentially. All association analyses were explicitly exploratory and unadjusted. No adjustment for multiple testing was made. Estimates were interpreted with attention to effect size, confidence-interval width, potential confounding and the number of comparisons. Non-significant findings were not interpreted as evidence of no association. Data were complete for all variables included in the reported analyses. All 90 participants were included, and no imputation was performed.

## Results

### Participant characteristics

Of 150 patients approached, 60 declined before eligibility assessment and 90 consented, were assessed as eligible and were included in the analysis (60.0%; Figure 1). None of the 90 was excluded, and no previous depression diagnosis was identified among them. Median age was 62.0 years (IQR 46.25–71.0; range 19–96), and 48 (53.3%) were female. Sixty-three (70.0%) had documented metastatic disease and 54 (60.0%) had at least one recorded comorbidity (Table 1).

**Table 1:** Baseline characteristics of the study participants (*N* = 90).

Characteristic	*n* or value	%
Age, median (IQR), years	62.0 (46.25–71.0)	—
Female sex	48	53.3
Male sex	42	46.7
Married	44	48.9
Current smoker	32	35.6
Education: low	11	12.2
Education: middle	12	13.3
Education: high	17	18.9
Education: university or above	50	55.6
Not employed	71	78.9
Cancer site: colon	20	22.2
Cancer site: breast	16	17.8
Cancer site: lung	13	14.4
Cancer site: blood	11	12.2
Cancer site: other	30	33.3
Metastatic disease	63	70.0
DNR status	72	80.0
Chemotherapy	68	75.6
Radiation therapy	32	35.6
Previous cancer surgery	21	23.3
Hormonal therapy	12	13.3
Immunotherapy	4	4.4
Any comorbidity	54	60.0
Hypertension	36	40.0
Diabetes	35	38.9
Cardiac disease	7	7.8
COPD	3	3.3
Asthma	4	4.4
Family history of psychiatric disease	26	28.9

Data were obtained through interviews and medical-record review and are presented as *n* (%) unless otherwise indicated. Treatment modalities and comorbidities are not mutually exclusive. Surgery refers to any prior cancer-related operation; other modalities include previous or ongoing treatments. Not employed means not currently holding a job and combines all reasons for non-employment; ability to work was not separately assessed. Individual cancer types and comorbidities are descriptive only. DNR, do-not-resuscitate; COPD, chronic obstructive pulmonary disease; IQR, interquartile range.

### PHQ-9 symptom burden

The median PHQ-9 score was 5.0 (IQR 4.0–10.75). Forty-three participants (47.8%) had none/minimal symptoms, 22 (24.4%) mild symptoms, 6 (6.7%) moderate symptoms, 9 (10.0%) moderately severe symptoms, and 10 (11.1%) severe symptoms. Forty-seven participants (52.2%; 95% CI 42.0–62.2%) scored ≥5, 25 (27.8%; 95% CI 19.6–37.8%) scored ≥10, and 19 (21.1%; 95% CI 14.0–30.6%) scored ≥15 (Table 2).

**Table 2:** PHQ-9 severity distribution and screening thresholds.

Measure	*n* or value	%	95% CI
Median total score (IQR)	5.0 (4.0–10.75)	—	—
None/minimal (0–4)	43	47.8	—
Mild (5–9)	22	24.4	—
Moderate (10–14)	6	6.7	—
Moderately severe (15–19)	9	10.0	—
Severe (20–27)	10	11.1	—
PHQ-9 ≥ 5	47	52.2	42.0–62.2
PHQ-9 ≥ 10	25	27.8	19.6–37.8
PHQ-9 ≥ 15	19	21.1	14.0–30.6
Item 9 ≥ several days	13	14.4	8.6–23.2

Data are presented as *n* (%) unless otherwise indicated. Severity categories are mutually exclusive and sum to 90; cumulative thresholds overlap. Confidence intervals are Wilson 95% intervals. The categories describe questionnaire scores, not diagnosed depressive disorders. PHQ-9, Patient Health Questionnaire-9; CI, confidence interval; IQR, interquartile range.

Fatigue or low energy was endorsed by 71/90 participants (78.9%), followed by appetite change in 65/90 (72.2%). Thoughts of death or self-harm were endorsed on at least several days by 13/90 (14.4%; 95% CI 8.6–23.2%). All 13 received same-day psychiatric assessment. Eight had no depression identified, two had delirium related to their medical condition, one had low mood related to the underlying medical condition, and two had depressive disorders. The clinical teams reported that no immediate suicide or self-harm risk was identified. The eight participants without depression required no further psychiatric intervention. Delirium was managed according to the relevant clinical protocol. The two participants with depressive disorders remained under psychiatric care; pharmacological treatment for depression was not initiated because their care prioritized comfort and minimizing interventions and medication burden near the end of life. These findings describe the immediate safety assessments and reported management, not longitudinal treatment outcomes. Item-level frequencies are shown in Figure 3.

**Figure 3:**
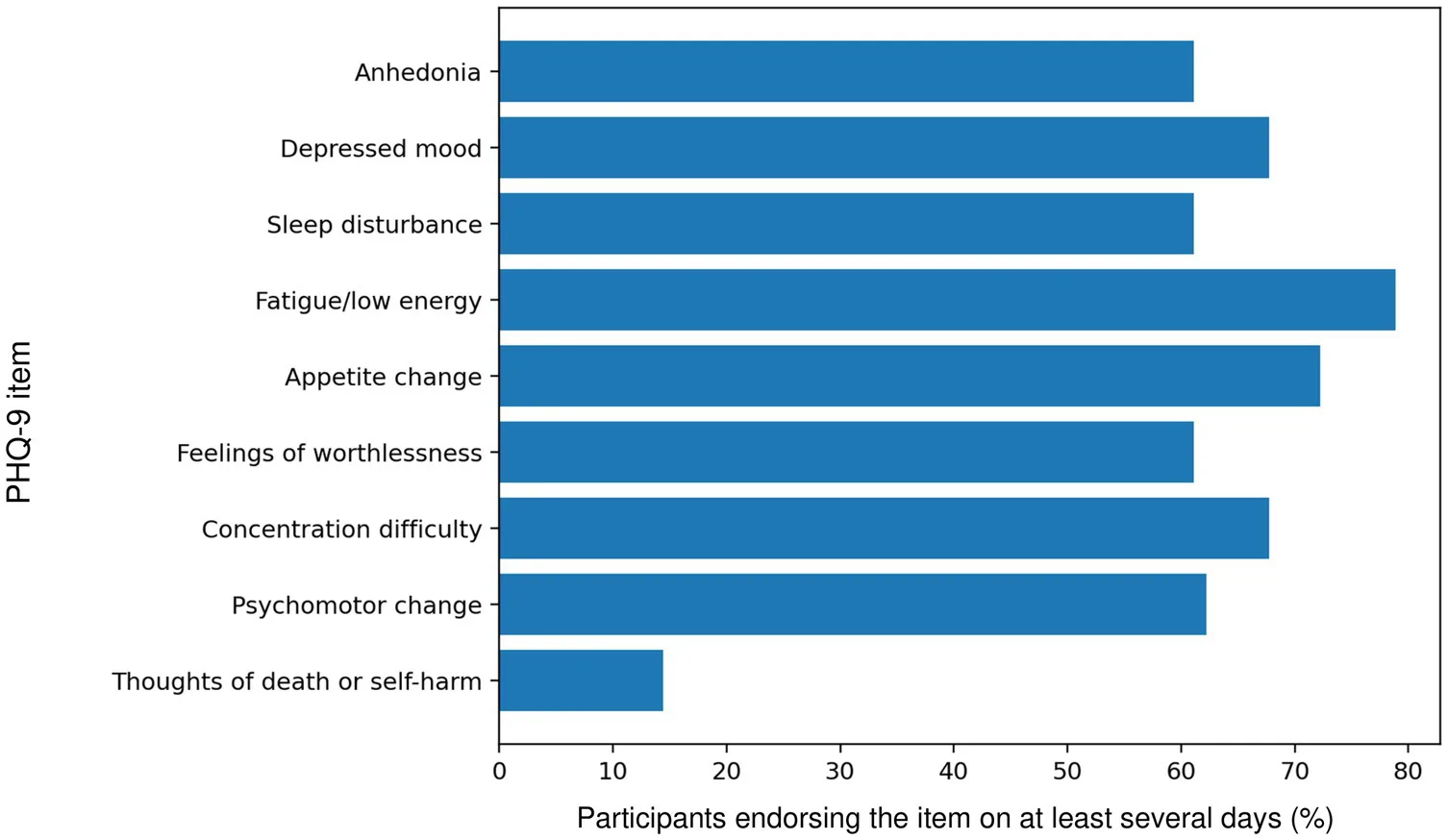
Frequency of endorsement on at least several days for each PHQ-9 item (*N* = 90).

### Exploratory comparisons by PHQ-9 threshold

Exploratory comparisons included 65 participants with PHQ-9 < 10 and 25 with PHQ-9 ≥ 10. All categorical OR confidence intervals included 1 (Table 3). Family history of psychiatric disease had an OR of 2.04 (95% CI 0.77–5.44), any comorbidity an OR of 1.61 (0.61–4.26), and employment an OR of 0.25 (0.05–1.15). These unadjusted estimates were imprecise and do not establish independent associations or the absence of an association. No multiple-testing adjustment was applied.

**Table 3:** Exploratory unadjusted comparisons by PHQ-9 screening threshold.

Characteristic	Reference category	PHQ-9 < 10 (*n* = 65)	PHQ-9 ≥ 10 (*n* = 25)	Unadjusted OR (95% CI)	Unadjusted *p*
Age, years; median (IQR)	Not applicable	62.0 (47.0–72.0)	62.0 (44.0–65.0)	—	0.404
Female sex	Male	37/65 (56.9%)	11/25 (44.0%)	0.59 (0.23–1.51)	0.347
Married	Not married	33/65 (50.8%)	11/25 (44.0%)	0.76 (0.30–1.93)	0.641
Current smoking	No current smoking	23/65 (35.4%)	9/25 (36.0%)	1.03 (0.39–2.69)	1.000
Employed	Not employed	17/65 (26.2%)	2/25 (8.0%)	0.25 (0.05–1.15)	0.083
Metastatic disease	Local disease	44/65 (67.7%)	19/25 (76.0%)	1.51 (0.53–4.34)	0.608
DNR status	No DNR status	52/65 (80.0%)	20/25 (80.0%)	1.00 (0.32–3.17)	1.000
Chemotherapy	No chemotherapy	50/65 (76.9%)	18/25 (72.0%)	0.77 (0.27–2.20)	0.785
Radiation therapy	No radiation therapy	24/65 (36.9%)	8/25 (32.0%)	0.80 (0.30–2.14)	0.807
Previous cancer surgery	No previous cancer surgery	18/65 (27.7%)	3/25 (12.0%)	0.36 (0.09–1.34)	0.165
Any comorbidity	No reported comorbidity	37/65 (56.9%)	17/25 (68.0%)	1.61 (0.61–4.26)	0.472
Family psychiatric history	No reported family history	16/65 (24.6%)	10/25 (40.0%)	2.04 (0.77–5.44)	0.195

All estimates and *p* values are exploratory and unadjusted for confounders or multiple testing. ORs compare the listed characteristic with its explicit reference category for the outcome PHQ-9 ≥ 10. Binary-variable *p* values use two-sided Fisher’s exact tests; age uses the Mann–Whitney *U* test. Employed is compared with all participants not currently holding a job, combined regardless of the reason. Individual cancer types and comorbidities were not tested. OR, odds ratio; CI, confidence interval.

## Discussion

### Principal findings

This study characterizes depressive symptom burden rather than diagnosed depression among patients receiving palliative oncology care. Approximately half of participants (52.2%) reported at least mild symptoms, while 27.8% reached the conventional PHQ-9 threshold of ≥10 for moderate-or-greater symptoms. Thirteen participants (14.4%) endorsed thoughts of death or self-harm and received same-day psychiatric assessment, emphasizing the clinical value of reviewing individual responses and linking screening to an immediate safety pathway rather than relying solely on the total score.

The item-level profile also illustrates a central interpretive challenge in palliative oncology. Fatigue or low energy and appetite change were among the most frequently endorsed symptoms, both of which can arise from advanced cancer and treatment as well as depression. The PHQ-9 therefore identifies symptom burden requiring clinical interpretation; it does not determine etiology or establish a depressive disorder (Grapp et al., 2019; Brown and Kroenke, 2009).

### Comparison with previous literature and study contribution

Direct comparison with previous studies requires attention to instrument, threshold, disease stage, care setting, country, and psychiatric-treatment eligibility. A recent Saudi palliative-care study used HADS and reported substantial depressive symptom burden, while another Saudi study of advanced cancer also used HADS and distress measures (Alotaibi and Alsuhail, 2025; Bakhsh et al., 2024). A more recent study in older Saudi adults receiving palliative oncology care incorporated PHQ-9 with patient activation and quality-of-life measures (Ibrahim and Zaghamir, 2026). International evidence likewise demonstrates substantial heterogeneity across self-report instruments and thresholds (Gontijo Garcia et al., 2023; Massie, 2004; Walker et al., 2013; Pitman et al., 2018; Mitchell et al., 2011). Against this background, the contribution of the present study is narrower but clinically specific: it provides a full PHQ-9 severity distribution, separates mild symptoms from the ≥10 screening threshold, reports individual symptom patterns, and specifically quantifies item 9 endorsement within a documented safety-referral pathway. This approach may improve early recognition of patients who warrant more detailed assessment, but the cross-sectional design does not demonstrate that screening itself prevents depression or improves outcomes.

### Potential bias

Of all patients approached, 60.0% participated. The 60 who declined were not assessed for eligibility, so their eligibility status and clinical characteristics were unknown. Reported reasons included lack of time, feeling well, perceiving no need to participate, or no reason provided; counts by reason were unavailable. These gaps prevent comparison of participants and non-participants and limit assessment of selection bias. The direction and magnitude of non-participation bias are uncertain.

Eligibility criteria excluding active psychiatric treatment and severe cognitive impairment may select a comparatively healthier or less psychiatrically burdened population and may underestimate symptom burden in the wider palliative-care population. No consenting patient was excluded, but the eligibility status of those who declined was unknown. Psychiatric treatment may also modify symptom scores. These effects cannot be quantified. Recruitment from a single service and the absence of separate outpatient and home-care counts further limit representativeness.

Interview-derived smoking, education, marital status, employment and PHQ-9 responses may be affected by recall or social-desirability bias. Medical-record review supported ascertainment of clinical variables but does not eliminate incomplete documentation or classification error. Family psychiatric history lacked information on the relative and disorder type, and specific cardiac diagnoses were not distinguished. The six-month definition of non-smoking may limit comparability with studies using other definitions.

### Exploratory associations

The exploratory comparisons were unadjusted and imprecise. None of the categorical OR confidence intervals excluded 1, but wide intervals remain compatible with clinically meaningful associations in either direction for several variables. Non-significant findings do not establish absence of an association.

### Clinical implications

The findings support careful case-finding rather than diagnostic labeling. In palliative oncology, a positive PHQ-9 screen should prompt review of symptom context, functional impact, patient preferences, and safety concerns. Item 9 requires particular attention because endorsement can indicate thoughts of death or self-harm but does not by itself establish suicidal intent. Early recognition may allow timely psychological or psychiatric assessment before distress becomes more severe, although prospective intervention studies are needed to determine whether structured screening and referral improve clinical outcomes. Screening for suicidal intention has also been evaluated in patients with cancer (Leung et al., 2013).

### Limitations

The PHQ-9 is a symptom-screening instrument rather than a diagnostic interview. The cited Arabic validation involved Saudi university students and does not establish the diagnostic accuracy or optimal cut-offs of this version in palliative oncology (AlHadi et al., 2017). The proportion scoring ≥10 represents moderate-or-greater depressive symptoms on screening, not the prevalence of clinically diagnosed depression, and must therefore be interpreted cautiously. Somatic overlap may elevate scores without a depressive disorder, while individual experiences of distress may not be fully captured by a total score. No standardized diagnostic interview was administered across the study cohort to resolve these uncertainties. Clinical psychiatric assessments were restricted to the 13 participants referred through the item 9 safety pathway and cannot validate screening thresholds for the full sample. Accordingly, the reported proportions represent screening symptom burden, not the prevalence of clinically diagnosed depression (Grapp et al., 2019; Kroenke et al., 2001).

The single-center cross-sectional design and modest sample limit generalizability and preclude causal inference. No formal prospective sample-size calculation was performed. Recruitment setting, functional status and detailed cancer symptom burden were unavailable at the participant level. Psychiatric assessments were performed for the 13 participants referred after item 9 endorsement, rather than as a standardized diagnostic evaluation of the entire cohort. Clinical follow-up continued as indicated by the treating teams; however, subsequent outcomes were not systematically collected for this study, so referral effectiveness and subsequent clinical outcomes could not be evaluated. The association analyses are vulnerable to confounding, and no adjustment for multiple testing was made. With only 25 participants scoring ≥10 and sparse categories, a multivariable model was not fitted because of the risk of overfitting and unstable estimates. These analyses should be considered hypothesis-generating.

## Conclusion

Approximately half of participants reported at least mild depressive symptoms, while more than one-quarter met the conventional moderate-or-greater PHQ-9 screening threshold. Thoughts of death or self-harm were reported by a notable proportion and triggered same-day psychiatric assessment. These findings characterize screening symptom burden, not diagnosed depression, and highlight the value of interpreting total scores together with individual symptoms and clinical context. Larger multicenter prospective studies incorporating diagnostic interviews, functional status, symptom burden and care setting are needed to determine whether structured screening and referral improve clinical outcomes.

## Data Availability

Study data are available from the corresponding author upon reasonable request and subject to institutional requirements.
